# Moving healthcare professionals – a whole system approach to embed physical activity in clinical practice

**DOI:** 10.1186/s12909-019-1517-y

**Published:** 2019-03-15

**Authors:** Michael Brannan, Matteo Bernardotto, Nick Clarke, Justin Varney

**Affiliations:** 1 Public Health England, London, UK; 2 Imperial College Healthcare NHS Trust, London, UK; 3 Public Health, Birmingham City Council, Birmingham, UK

**Keywords:** Whole system approach, Medical education, Physical activity, eLearning

## Abstract

**Background:**

Healthcare professionals are key informants to support individual behaviour change, and although there has been some progress in empowering clinicians to promote physical activity and health at work, an effective strategy overarching the whole medical educational journey is still lacking. This report provides an overview from the Moving Healthcare Professionals programme (MHPP), a whole-system educational approach to embed prevention and physical activity promotion into clinical practice.

**Methods:**

The MHPP model integrates educational resources into three core domains of medical education: undergraduate education, postgraduate education and continuing professional development. The interventions are designed to spiral through existing educational approaches rather than as additional special study modules or bolt-on courses, thus reducing self-selection bias in exposure. Interventions include spiral undergraduate education materials, e-learning, embedded post-graduate resources and face-to-face peer-to-peer education.

**Results:**

To date the MHPP model has been applied in two key areas, physical activity and health and work. The physical activity programme in a partnership between Public Health England and Sport England has delivered face-to-face training to 17,105 healthcare professionals, embedded materials in almost three quarters of English medical schools and overseen > 95,000 e-learning modules completed over two and half years. Evaluation of the individual elements of the model is ongoing and aims to show improvements in knowledge, skills and practice. Further evaluation is planned to assess patient impact.

**Conclusions:**

The MHPP model offers a coherent whole-system approach to embed public health action into existing healthcare education models, and as such provides a framework for rapid change as well as upstream implementation to support the clinicians of today and tomorrow.

## Background

There is substantial evidence supporting the positive impact of behavioural interventions to promote physical activity and healthy lifestyle [1]; however, there is also a need for more effective programmes and collaborations [2]. At present, healthcare professionals appear to be struggling to deliver these messages, and response from patients has been mixed [3]. In fact, both adult and older patients often feel they are not receiving physical activity interventions or guidance from their general practitioners (GPs) [4, 5].

Research has shown that primary healthcare professionals such as GPs already frequently discuss physical activity with their patients, although this advice is typically brief, non-specific and therefore fails to capitalise on the opportunity to change patients’ behaviour and engagement with a more active lifestyle [6]. However, even within medical specialities there is a significant individual inter-clinician variability on how exercise and physical activity is prescribed to specific patient groups, for instance with cardiovascular comorbidities, which highlights the lack of standardisation and recognised guidance [7]. Furthermore, interventions are generally on a single encounter basis and are often inadequate in driving change in physical activity engagement and function [8]. There is therefore a great need for both GPs and physicians to be effectively trained to promote physical activity and prescribe exercise [9, 10]. Recent surveys found that many physicians, including newly qualified doctors, lack the confidence to deliver physical activity counselling; and despite changes in national guidelines in the United States (US), counselling behaviour has not increased [11]. At undergraduate level, in English medical schools there is widespread lack of training on physical activity promotion [12]. Among other healthcare professionals such as physiotherapists, research has shown some improvement in their understanding of physical activity counselling [13], but there is still generalised poor understanding [14] and wide-spread unawareness about the current physical activity recommendations [15].

On the other hand, patients may have unrealistic expectations about what healthcare professionals can deliver in terms of physical activity counselling, which in itself is a significant barrier to successful intervention [16]. In primary care this barrier to integrate physical activity promotion is compounded by reported lack of time, skills, cost reimbursement, as well as adequate screening [17]. In addition, the lack of co-ordinated large scale initiatives reduces the impact of any intervention. There is a need not just for better community-based collaborations with sport, leisure and fitness providers [18], but also the improved infrastructure, such as access to walking or biking, that was shown to define effective population-level interventions [19].

Despite the challenges physical activity promotion remains a cost effective and viable option of health promotion and prevention [20], and in fact, primary care work force and GPs are probably the most cost-effective healthcare professionals to deliver physical activity counselling [21]. However, attitudes and behaviours among healthcare professionals are influenced from the early career stages, as the confidence in delivering physical activity interventions among medical students and doctor is associated with their own personal exposure and engagement with physical activity [22]. In order to address the specific needs of different healthcare professionals, the Moving Healthcare Professional Project (MHPP) was developed as an overarching model capturing all stages of medical education, from undergraduate and postgraduate to continuing medical education. It has been shown to have the potential to drive and embed cultural changes within the health professions that could significantly improve the quality of physical activity counselling and promotion [23, 24]. This study looks at the early implementation of the MHPP in the England and its ongoing development.

## Methods

### The moving healthcare professional Programme (MHPP)

**Fig. 1:**
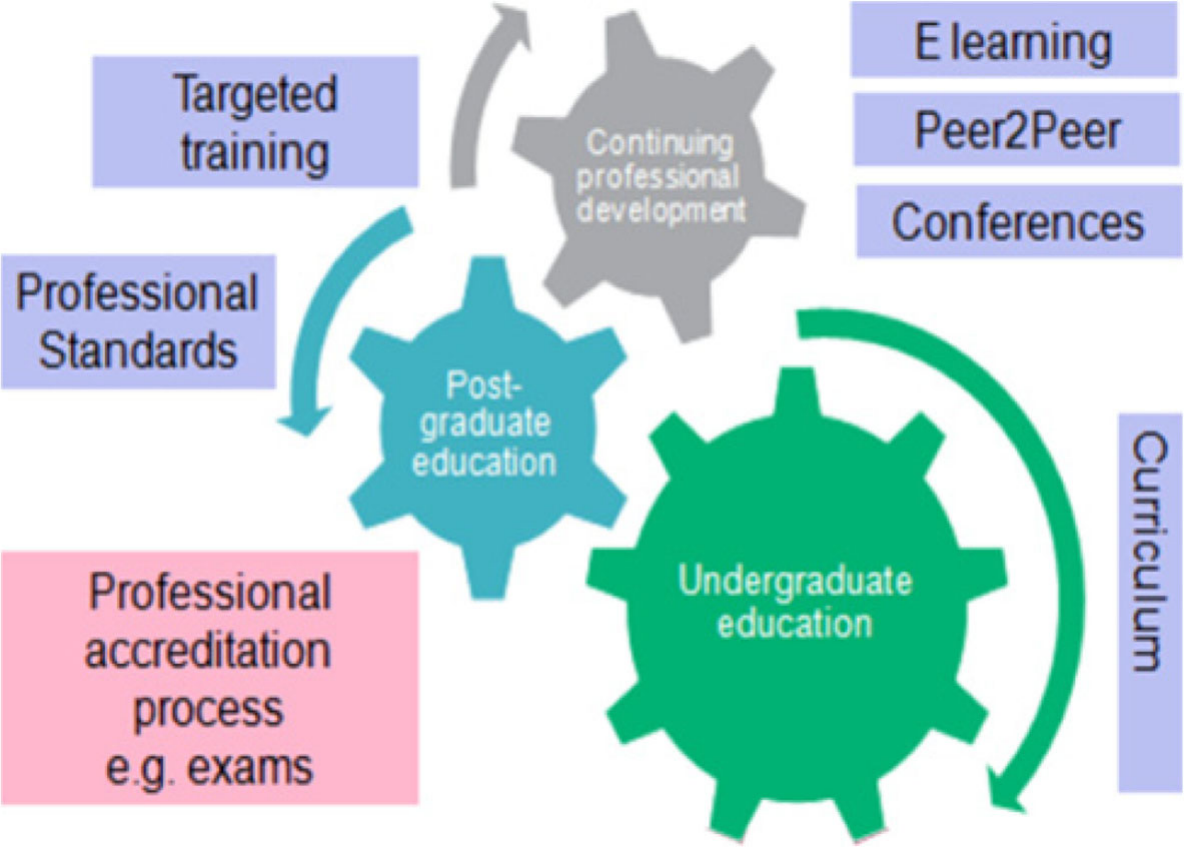
“Moving Professionals – The model”. Cog diagram showing the overview of the MHPP

The MHPP is based on a simplified pathway of medical education, where individuals move from undergraduate education into a period of post-graduate structured training and education, followed by ongoing continuing professional development (CPD) once working within a defined specialism. Within each of these three stages there are structured educational components (e.g. undergraduate curriculum and teaching resources), and the MHPP model was developed to embed within these components (Fig. 1).

The programme model was initially developed for physical activity through a series of workshops with key national stakeholders, including medical school deans, medical royal colleges and other healthcare professional bodies, medical trade unions and front line clinicians. This facilitated the development of specific peer-to-peer training and e-learning modules to deliver the MHPP. Some components of the programme were already in development for other projects, such as the spiral medical undergraduate teaching materials on physical activity, so there was a synergistic opportunity to integrate them as the MHPP evolved. The programme was initially piloted in one region (London) and then expanded nationwide throughout England for physical activity over two years; this approach was then mirrored for health and work.

### The physical activity MHPP

The initial pilot for the MHPP was focused on delivering physical activity education to doctors in partnership with Sport England. An overview of programme’s development timeline is shown in Table 1. The programme has four core components:

1. Undergraduate spiral education materials

**Table 1:** BMJ eLearning modules usage from January 2014 to April 2018

Module title		Started	Completed	Completion ratio
1	Importance of physical activity	14,544	13,611	94%
2	How physical activity produces health benefits	10,001	9435	94%
3	The health Benefits of physical activity: cancer	12,108	11,576	96%
4	The health benefits of physical activity: cardiovascular disease	11,517	10,839	94%
5	The health benefits of physical activity: depression, anxiety and sleep	11,595	10,763	93%
6	The health benefits of physical activity: diabetes	15,586	14,548	93%
7	The health benefits of physical activity: osteoarthritis and low back pain	10,924	10,418	95%
8	The health benefits of physical activity: promoting physical activity in primary care	9484	8868	94%
9	The health benefits of physical activity: respiratory disease	9555	9077	95%
**Total**		**105,314**	**99,135**	**94%**

The spiral teaching resources are a suite of 22 slides and teaching notes that can be integrated into core curriculum teaching, (i.e. slides on physical activity and hypertension can be integrated into a general lecture on hypertension). The slides were developed by an international collaboration of medical educationalists and academics and validated by the Council of Medical School deans. Alongside the teaching materials a suite of 150 multiple-choice questions were also developed. The resources are free to use in England and are managed by “Exercise Works”.

2. Postgraduate embedded educational resources

This element has been developed and led by the Faculty of Sport and Exercise Medicine (FSEM), providing interactive educational materials tailored to different clinical specialisms and embedding them within the relevant Medical Royal College website and curricula.

3. Continuing professional development e-learning resources

A suite of nine e-learning modules were developed on the British Medical Journal (BMJ) e-learning platform chosen because of its unique reach to doctors and its credibility as a medical CPD provider. Alongside the core modules an additional module on motivational interviewing techniques was also developed.

4. Face to face peer educators (clinical champions)

Clinical Champions are healthcare professionals who are paid on a sessional basis to deliver a standardised training package which has been peer reviewed and sponsored by Public Health England (PHE). Clinical champion training is delivered directly to healthcare professionals and where possible the standardised package is supplemented with information on the local physical activity programmes to encourage signposting.

In parallel to the core programme components, there was also work to develop the Chief Medical Officers guidelines on physical activity at different lifecourse stages into infographics which were integrated into the educational resources and disseminated as supplements in the BMJ, an article in the British Journal of Sports Medicine (BJSM), conferences, media events and press releases.

### Ethics

The MHPP was approved by Public Health England Research Ethics Committee (PHE REC). The data presented in this manuscript was collected after obtaining informed written consent from all participants that completed eLearning modules and/or face-to-face training as part of the MHPP.

## Results

### Timeline

**Fig. 2:**
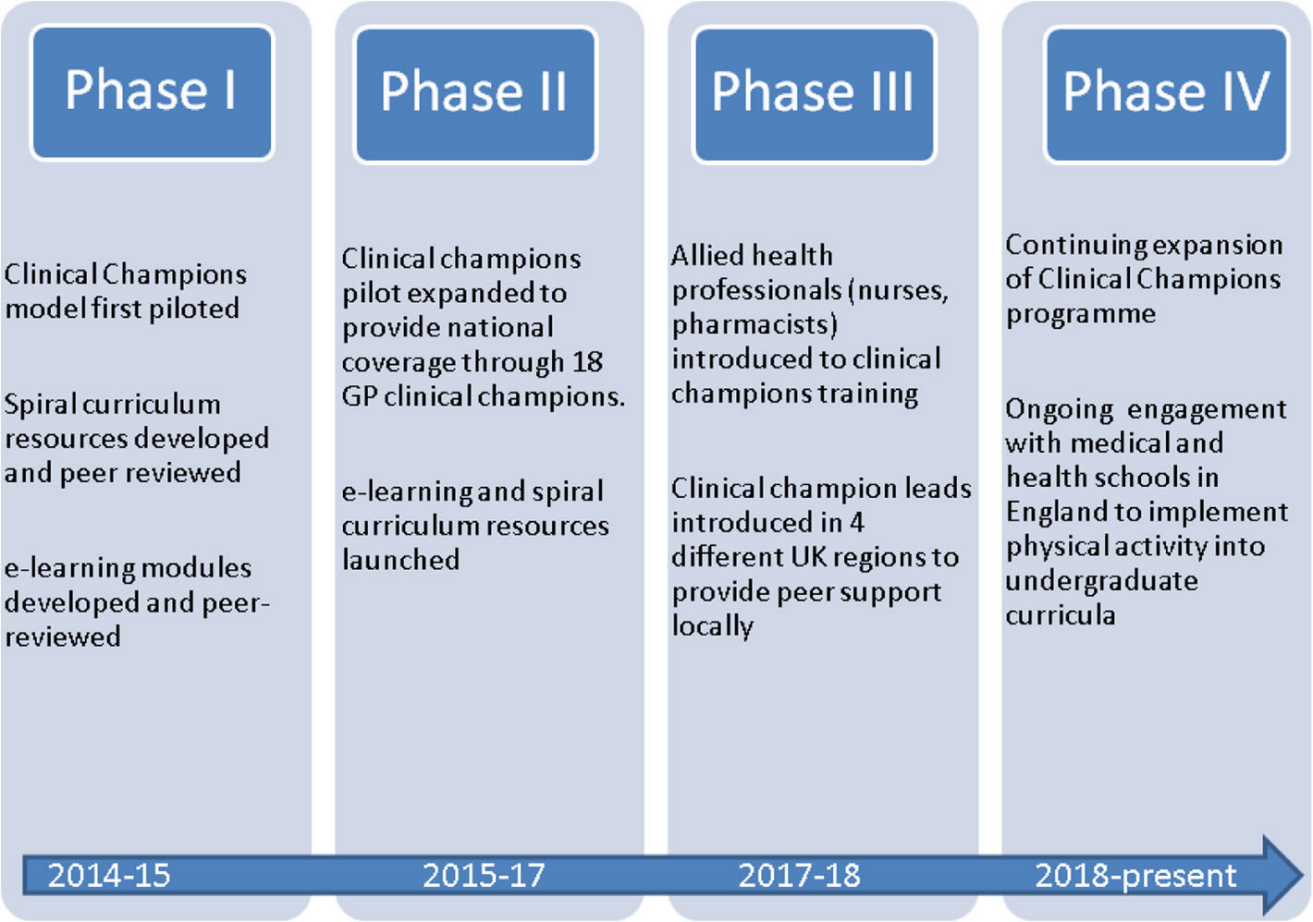
“Timeline”. MHPP development timeline

Since its first implementation in 2014 the MHPP has significantly expanded and addressed the four core components of the physical activity modules. When the *Clinical Champions* model was initially piloted, a single GP trained as a clinical champion was funded to deliver physical activity training using a standardised set of teaching slides. Since then, the Clinical Champions have grown in number and continue to deliver training to nationwide to doctors, nurses and allied healthcare professionals. In parallel, spiral curriculum resources developed by “Exercise Works” and Nottingham Medical School were gradually implemented across medical school curricula. Furthermore, a suite of 9 e-learning modules to deliver CPD were developed and peer-reviewed for the BMJ e-learning platform. A chronological overview of the programme’s development and implementation is shown in Fig. 2.

### Undergraduate education

A target of 35 English medical or health schools has been commissioned by PHE to achieve the MHPP’s undergraduate education goals. To date, 26 (74%) medical schools have agreed to implement physical activity modules and education into undergraduate curricula or have done so already. Further evaluation is being undertaken to understand the barriers and enablers for integration of the resources, and whether those not actively using the resources have alternate similar material already embedded across their curricula.

### eLearning resources

In collaboration with BMJ Learning an online course on physical activity in the treatment of chronic condition was launched in October 2014 [25]. The course comprises 9 separate modules covering physical activity for different conditions, from mental health to diabetes and cancer, totalling 4 and half hours of recognised CPD activity. Since its inception > 95,000 eLearning modules have been completed (Table 1) by healthcare professionals from diverse specialisms and professional groups, despite the platform being primarily targeted at doctors. Qualitative feedback on the modules from participants through the platform feedback survey has been very positive.

### Postgraduate face-to-face education

Postgraduate education has been driven by the *Clinical Champions* programme, where peer training on physical activity counselling and exercise prescription is delivered directly to practicing healthcare professionals in the form of recognised CDP modules. This branch of the MHPP has impacted to date an estimated 17,105 healthcare professionals (Table 2) and there has been good geographical coverage as there has been a core of champions delivering training in each of PHE’s nine regions. Although it is not possible from the routine data collection to judge how much overlap there is between different strands of the programme we would expect there to be a strong element of cross-pollination with the e-learning materials to reinforce the face to face teaching.

**Table 2:** Activity data on Programme Strands, (HCA healthcare Assistant, HCP healthcare professional)

Phase	Number trained	Detailed audience breakdown^a^
Phase 1–2014/15	217	217 GP trainees
Phase 2–2015/16	5156	2199 GPs 1738 Other doctors 1132 Nurses 87 HCAs and other HCPs
Phase 3–2016/17	4959	2041 GP 1386 Trainee GP 1380 Other HCP 152 Trainee other HCP
Phase 4–2017/18*	6773	2381 GPs & HCPs 1273 Trainee GPs & HCPs 3119 Nurses
Grand total	17,105	

^a^up to end of February 2018

The evaluation of the clinical champion’s programme is currently being undertaken. However, in line with the results from the initial pilot, it should hopefully be able to demonstrate a positive impact on clinicians’ knowledge about physical activity in both primary and secondary prevention, as well as more confidence in integrating brief advice on physical activity into routine consultations.

## Discussion

Considering the World Health Organisation (WHO) predicts that by 2020 two thirds of disease burden worldwide will be due to poor lifestyle choices [26], interventions to introduce lifestyle medicine teaching into undergraduate and postgraduate health education are expected to have a significant public health impact [27] and are in line with the WHO global action plan on physical activity (GAPPA) to reduce the prevalence of sedentary lifestyles [28]. In this context, the MHPP model is a coherent, co-produced whole system educational approach to embed effective physical activity counselling into routine clinical training and ultimately everyday clinical practice. It presents a viable and transferrable model for change that can be replicated for different topics and challenges, and its use of different learning styles and preferences across the professional “lifespan” ensures comprehensive coverage. Ultimately, the MHPP provides a framework for change that we hope will underpin a step change in the approach to physical activity in clinical practice in England for today and tomorrow’s healthcare professionals.

MHPP was designed to take a whole educational system approach to embed physical activity into clinical practice, including the upstream integration in undergraduate education as well as the downstream capability development with qualified healthcare practitioners. The core interventions focused on undergraduate, postgraduate and peer-to-peer education as well as eLearning, which were chosen as no single educational approach used in isolation has been shown to provide effective and enduring changes among healthcare professionals [7, 8, 11]. These core elements were supported by a consistent public health awareness campaign reiterating the importance of physical activity advice from clinicians. By taking this “whole educational life” approach, the model is designed to eventually become redundant as the clinical social norm shifts toward the routine integration of brief advice on physical activity into daily clinical practice for all clinicians in England.

The MHPP’s implementation of spiral education into undergraduate curricula addresses the inconsistent education on physical activity counselling and exercise prescription that healthcare students receive [12, 22]. In addition, the postgraduate curricula changes driven by FSEM also address the perceived lack of adequate training among trainee doctors, who report poor knowledge, skills and competence to provide patient-centred exercise counselling and exercise prescription [29]. Spiral integration was chosen as an established approach used successfully to embed a common message across multiple different topics and learning experiences [30], reducing the risk that the message only reaches those who are already motivated, thus continually reinforcing the benefits of physical activity across multiple disease conditions and in the context of both prevention and treatment of disease.

The inclusion of a large eLearning component is in line with previous research supporting the use of this modality among medical students, where a large meta-analysis of 59 studies showed equivalent satisfaction and effectiveness when compared with traditional teaching methods [31], and also reflects the growing trend to deliver postgraduate medical education for busy healthcare professionals through e-learning. The MHPP builds on this with a multi-faceted model that implements eLearning alongside face-to-face training, which has been shown in different studies to be both practical and cost-effective in helping healthcare professionals deliver a self-management support programme and physical activity counselling to chronically ill patients [32–34]. The peer education ‘champions’ focused on delivering training through existing programmed teaching time which was especially effective in reaching GPs but also provided a recognisable context for CPD training. This approach was further strengthened by having ‘peer educators’ who could relate the theory to practical case studies and share tips and hints for the practical application of the evidence. The use of trained healthcare professionals (“Clinical Champions”) to deliver capillary training on physical activity counselling and prescription is not only a novel approach built on the best evidence available, but also a strategy that addresses the limitations of models based solely on eLearning [35].

Taking a whole educational system approach to embed cultural change in medical education is complicated and requires strategic horizon scanning as well as well-resourced implementation and delivery. In addition, like all novel interventions, it comes with challenges as well as limitations. The MHPP has benefited from having PHE, the national public health agency responsible for England, to act as pivot between all key national partners involved in developing a whole new educational system. Nonetheless, a public health body in isolation is not sufficient in itself to deliver these wide-ranging changes, and the MHPP represents a feasible and synergistic co-production model where other healthcare professional organisations, trade unions and education bodies collaborate toward a shared goal maintaining high and consistent standards across the board. The use of established stakeholders such as the BMJ Group provided resources and platforms with high penetration and across all medical specialisms in England. The wide reach of BMJ publications, as well as recognised CPD accreditation, explains some of the high uptake and completion of eLearning modules, and we understand that using such platforms may not be feasible in every location, although it emphasises the advantage of using established media rather than radically new dissemination platforms.

In terms of evaluating the effectiveness of the programme, the focus is on its impact on confidence and capability to provide brief advice, as there is already a clear and consistent evidence base supporting brief advice from healthcare professionals on physical activity leading to improved rates of physical activity uptake in patients [36]. However, issues of objectivity and impartiality in the evaluation could arise when a single agency oversees the process. To minimise this bias, the evaluation of the MHPP has been delegated to independent academics at the University of Loughborough and Sheffield Hallam University, so we are therefore unable to conclude on the effectiveness of the MHPP. The challenge of evaluating multiple interventions across different stakeholders remains, and this complexity may yet affect the validity of the evaluation. Yet the evaluation will add to our understanding of the optimal educational model, shed light on the interconnectivity between components of the programme, the differential impact with different groups of clinicians and specific barriers emerging.

## Conclusions

This technical paper sets out a novel whole system educational approach to embed routine brief advice on physical activity into every day clinical practice. The approach used could provide a model for future public health actions aiming to promote cultural changes and shift educational paradigms. However, future success will depend on objectively recognising, evaluating and addressing the barriers and facilitators to the implementation process. The initial engagement with the programme has been significant but time and further analysis will tell if the upstream implementation of the programme is achieving its goal of major cultural change among practitioners and the ultimate outcome of supporting more people to get active every day in England.

## Abbreviations

BMJ: British Medical Journal
CPD: continuing professional education
FSEM: Faculty of Sports & Exercise Medicine
GP: general practitioner
HCA: healthcare assistant
HCP: healthcare professional
MHPP: Moving Healthcare Professional programme
PHE: Public Health England
